# Results of the first-in-human, randomized, double-blind, placebo-controlled, single- and multiple-ascending dose study of BIIB113 in healthy volunteers

**DOI:** 10.1016/j.tjpad.2025.100302

**Published:** 2025-07-21

**Authors:** Flavia C. Nery, Maciej Kaliszczak, Ben Suttle, Lori Jones, Shuang Wu, Jing Xie, Gioacchino Curiale, Esin Yesilalan, Beth Hirschhorn, Denisa Wilkes, Dave Singh, Martin Bolin, Sangram Nag, Andrea Varrone, Per Stenkrona, Anton Forsberg Morén, Christer Halldin, Jeffrey Yachnin, H. Moore Arnold, Szofia Bullain, Jaren Landen, Diana Gallagher, Heike Hering

**Affiliations:** a Biogen, Cambridge, MA, USA; b Biogen, Durham, NC, USA; c qPharmetra, Raleigh, NC, USA; d Hammersmith Medicines Research Limited, London, UK; e Medicines Evaluation Unit, The Langley Building, Manchester University NHS Foundation Trust, University of Manchester, Manchester, UK; f Department of Clinical Neuroscience, Center for Psychiatry Research, Karolinska Institutet, Stockholm, Sweden; g Karolinska Comprehensive Cancer Center, Huddinge, Sweden

**Keywords:** Alzheimer’s disease, Tau, BIIB113, O-GlcNAcase, Target occupancy

## Abstract

**Background:**

Preclinical studies have demonstrated that inhibition of the O-linked β-N-acetylglucosaminidase enzyme increases tau O-linked β-N-acetylglucosaminylation and may attenuate tau pathology in Alzheimer’s disease.

**Objectives:**

To examine the safety, tolerability, pharmacokinetics, and target occupancy of single- and multiple-ascending oral doses of the small-molecule O-linked β-N-acetylglucosaminidase inhibitor, BIIB113.

**Design:**

Study 276HV101 was a first-in-human, multicenter, Phase 1, randomized, double-blind, placebo-controlled, single- and multiple-ascending dose trial.

**Setting:**

72 participants were enrolled from February 2022 through July 2023.

**Participants:**

Adult healthy female and infertile/vasectomized male participants.

**Intervention:**

In the single-ascending dose substudy, participants received a single dose of placebo or BIIB113 0.5, 3, 15, or 50 mg. In the 14-day multiple-ascending dose substudy, participants received placebo or BIIB113 15 or 50 mg once daily. In the target occupancy substudy, participants received either a single dose of BIIB113 0.5 or 3 mg or a once-daily dose of BIIB113 0.5 mg.

**Measurements:**

Safety and tolerability were measured by incidence of adverse events. Pharmacokinetic and concentration-time profiles were assessed. Target occupancy was evaluated using the carbon-11 BIO-1819,578 radioligand.

**Results:**

BIIB113 was generally well tolerated. Pharmacokinetics were linear over the dose range, with a half-life of approximately 30 h. Administration with food decreased the rate but did not affect the extent of absorption. There were no clinically meaningful differences in pharmacokinetics between elderly and nonelderly participants. Multiple once-daily doses of BIIB113 0.5 mg maintained a target occupancy of ≥90 % up to 48 h.

**Conclusions:**

BIIB113 was well tolerated and achieved high levels of target occupancy.

## 1 Introduction

In Alzheimer’s disease (AD), accumulation of amyloid plaques and hyperphosphorylated neurofibrillary tangles (NFTs) underlie the progressive decline in cognition and behavior [1,2]. AD is considered to be an amyloid-driven secondary tauopathy in that amyloid is thought to drive the accumulation of NFTs in a distinct spatiotemporal pattern as the disease progresses [3]. The progression of tau pathology across brain regions, which can be measured by tau positron emission tomography (PET), correlates well with the neurodegeneration and cognitive and functional decline in AD [3,4].

Reduced O-linked β-N-acetylglucosaminylation (O-GlcNAcylation) of tau provides a link between glucose hypometabolism and the aggregation of tau into NFTs during AD [5]. In patients with AD, cerebral glucose hypometabolism, measured by fluorodeoxyglucose-18-PET, correlates with tau deposits measured by tau PET, and glucose hypometabolism and the regional distribution of tau deposits correlate well with symptom severity [6,7]. The O-linked β-N-acetylglucosamine (O-GlcNAc) modification, which happens frequently in neurons, is a nutrient-responsive posttranslational modification of neuronal proteins [5]. O-GlcNAc levels are coupled to glucose availability through the action of the enzymes O-GlcNAc transferase and O-GlcNAcase (OGA). O-GlcNAc transferase conjugates O-GlcNAc onto tau proteins, and OGA catalyzes the removal of O-GlcNAc from tau (**Supplemental Figure 1**) [8,9]. O-GlcNAcylation is thought to play a role in the development of tau pathology in AD because of its ability to attenuate tau aggregation [5,10].

Decreased brain O-GlcNAcylation is observed in AD, which suggests that glucose hypometabolism may impair the protective roles of O-GlcNAc within neurons and enable tau aggregation and neurodegeneration [5,11]. Studies in transgenic tau pathology mouse models have demonstrated that OGA inhibition increases tau O-GlcNAcylation and reduces tau pathology [[11], [12], [13], [14], [15], [16], [17]]. Together, these findings and the striking regional overlap of glucose hypometabolism and tau deposition in AD brains suggest that inhibition of the OGA enzyme is a potential therapeutic approach to target tau accumulation in AD.

BIIB113 is an oral small-molecule inhibitor of OGA intended to reduce the progression of tau pathology through increased tau O-GlcNAcylation and attenuated aggregation [18], which is hypothesized to slow the cognitive decline seen in AD [5]. BIIB113 was shown to reduce the formation of tau pathology in a mouse model of tau pathology [18].

The present study evaluated the safety, tolerability, pharmacokinetics, and target occupancy of BIIB113 in a randomized, double-blind, placebo-controlled, single- and multiple-ascending dose (SAD and MAD, respectively) Phase 1 trial in healthy volunteers. To analyze target engagement, OGA-PET was performed after single and multiple doses of BIIB113. Target occupancy was evaluated using the OGA-PET tracer carbon-11 (^11^C) BIO-1819,578 [10,19].

## 2 Methods

### 2.1 Study design

**Fig. 1:**
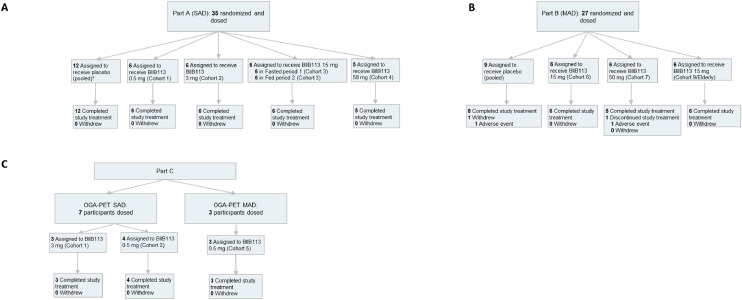
Patient flow and dosing schema during the SAD, MAD, and OGA-PET SAD substudies.

Study 276HV101 (NCT05195008) [20] was a first-in-human, multicenter, Phase 1, randomized, double-blind, placebo-controlled, SAD and MAD trial evaluating the safety and pharmacokinetics of BIIB113 in healthy adult participants. The patient flow and dosing schema are shown in Fig. 1. Parts A (SAD substudy) and B (MAD substudy) were randomized, double-blind, placebo-controlled studies assessing the safety, tolerability, and pharmacokinetics of BIIB113. Part C was a single-center, open-label substudy to determine brain target occupancy of BIIB113 using [^11^C]BIO-1819,578 [10,19].

Participants in the SAD/MAD cohorts were dosed consecutively, with 6 participants on active dose and 3 on placebo in each cohort. All participants were monitored closely in the patient unit, and a safety medical review was conducted at the completion of each cohort to make dose-escalation decisions. Cohort 8, the highest dose cohort (100 mg) in the Part B MAD substudy, was not initiated as the dose was refined based on the target occupancy readout from Part C (OGA-PET SAD substudy). * Three participants from Cohort 3 (fed, Period 2) received a second oral dose of placebo, separated from the first dose by a washout period of at least 2 weeks. All 3 participants completed the study treatment, and none withdrew. MAD, multiple-ascending dose; OGA, O-GlcNAcase; PET, positron emission tomography; SAD, single-ascending dose.

This study enrolled healthy female and infertile/vasectomized male participants aged 18 to 64 years in Parts A (SAD) and B (MAD) (Part B also contained an elderly cohort of individuals aged 65 to 75 years) across 2 sites. Part C (OGA-PET SAD/MAD substudy) enrolled healthy participants aged 20 to 64 years at a single site.

In Part A (SAD substudy), 35 participants were randomized across 4 dose-ascending cohorts; within each cohort, participants were randomized 6:3 to receive 1 of 4 doses of BIIB113 (0.5, 3, 15, or 50 mg) or placebo. In Part B (MAD substudy), 27 participants were randomized 6:3 across 3 cohorts (2 cohorts of nonelderly healthy adults and 1 cohort of elderly healthy adults) to receive BIIB113 or placebo. Nonelderly participants received placebo or BIIB113 15 mg or 50 mg orally once daily (QD) for 14 days. The elderly participants randomized to BIIB113 received a 15-mg dose QD for 14 days. In Part C (OGA-PET substudy), 7 participants received a single oral dose of BIIB113 0.5 or 3 mg (SAD), and 3 participants received BIIB113 0.5 mg QD for 14 days (MAD).

The trial was conducted in accordance with the Declaration of Helsinki and all applicable International Council for Harmonisation and Good Clinical Practice guidelines. Investigators obtained institutional review board or ethics committee approval before beginning the study. All participants provided written informed consent before participating in any study-related activities.

### 2.2 Study objectives

The primary objective of the study was to assess the safety, tolerability, pharmacokinetics, and target occupancy of single- and multiple-ascending oral doses of BIIB113 in healthy participants. Secondary objectives included an evaluation of the single and multiple oral-dose pharmacokinetic profile of BIIB113 in healthy participants and an evaluation of the effect of food on the single oral-dose pharmacokinetic profile of BIIB113 in healthy participants. Exploratory objectives included assessment of the effect of BIIB113 on corrected QT (QTc) interval and other electrocardiogram (ECG) parameters and an evaluation of the potential induction of cytochrome P450 3A (CYP3A) activity by BIIB113 in healthy participants.

### 2.3 Fed and fasting conditions for the SAD cohort

The effect of food on the pharmacokinetics of a single dose of BIIB113 was investigated in participants enrolled in Cohort 3 of Part A (SAD). In Period 1 of Cohort 3, participants received BIIB113 15 mg or placebo under a fasted condition. Period 2 (fed condition) followed after a washout period of ≥2 weeks, during which participants received the same treatment as in Period 1 (after an overnight fast of ≥8 h, which did not include water, and within 30 min after starting a high-fat, high-calorie breakfast). A high-fat (approximately 50 % of total caloric content of the meal) and high-calorie (approximately 800 to 1000 kcal) meal was provided that derived approximately 150, 250, and 500–600 kcal from protein, carbohydrate, and fat, respectively.

### 2.4 Target occupancy of BIIB113 measured by OGA-PET

For participants enrolled in Part C, the target occupancy of OGA by BIIB113 was assessed using an OGA-PET tracer [^11^C]BIO-1819,578 [10,19]. Each participant received a baseline PET scan with no administration of BIIB113 following the intravenous administration of 194 ± 35 megabecquerels of [^11^C]BIO-1819,578. Participants received a predetermined oral dose of BIIB113 and then another IV dose of [^11^C]BIO-1819,578, and they were scanned again after an early (between 2 and 6 h after BIIB113 administration) and late (between 24, 48, or 72 h after BIIB113 administration) time point.

PET acquisition was performed for up to 93 min, and arterial blood was collected using an automatic blood sampling system followed by discrete blood samples. Radiometabolite analyses were performed using reverse-phase radio-high-performance liquid chromatography [21] to establish the parent fraction. Correcting the radioactivity measured in blood with the parent fraction yielded the arterial input function. Volumes of distribution (V_T_) were estimated using Ichise’s multilinear analysis model [22] with arterial input function and were calculated for baseline measurements and posttreatment with BIIB113. Up to 3 administrations of the tracer [^11^C]BIO-1819,578 and 3 PET scans were obtained for each participant. The difference between the baseline and the postdose V_T_ at 2 time points was calculated using a revisited Lassen plot to determine target occupancy in a dose- and time-dependent manner. Plasma pharmacokinetics were assessed at multiple time points up to 72 h after BIIB113 administration.

### 2.5 Statistical analyses

#### 2.5.1 Primary endpoints

Adverse events (AEs), severe AEs (SAEs), clinical laboratory abnormalities, vital signs, and ECGs were summarized using frequency tables for categorical outcomes and descriptive statistics for continuous outcomes by treatment and dose level. Columbia Suicide Severity Rating Scale (C-SSRS) data were summarized using descriptive statistics (i.e., the number of participants, mean, standard deviation [SD], median, minimum, and maximum) for continuous variables and using frequency and percentage for discrete variables. For the 15-mg dose level in Part A, the data were summarized by fasting versus fed conditions. In Parts A and B, placebo data across cohorts were pooled, while in Part C, target occupancy was summarized by treatment group and time.

#### 2.5.2 Secondary endpoints

Descriptive statistics were evaluated for the pharmacokinetic parameters and plasma concentrations of BIIB113. Mean concentration-time profiles were plotted on linear and semi-logarithmic scales. The dose proportionality of BIIB113 after administration of a single dose (Day 1 of the SAD substudy) was descriptively assessed based on area under the concentration-time curve from time zero to the time of the last measurable concentration (AUC_last_), AUC from time zero extrapolated to infinity (AUC_inf_), and maximum observed concentration (C_max_) using a power model. The estimated slope and 95 % confidence interval (CI) were calculated. Dose proportionality of BIIB113 after administration of multiple doses (Days 1 and 14 of the MAD substudy) was assessed descriptively based on AUC_last_, AUC within a dosing interval (AUC_tau_), and C_max_.

To estimate the effect of food on BIIB113 single-dose pharmacokinetics (Part A SAD), the log-transformed AUC_last_, AUC_inf_, and C_max_ from Day 1 of the fasting and fed periods were analyzed using a mixed-effect model, with treatment (fasted or fed) as a fixed effect and participant as a random effect. Estimates of the adjusted mean differences and corresponding 90 % CI were obtained from this model. The adjusted mean differences and 90 % CIs for the differences were exponentiated to provide estimates of the ratio of adjusted geometric means and 90 % CI.

To assess the relationship between OGA target occupancy and plasma concentrations of BIIB113, a simple maximum effect (E_max_) model was fitted to the OGA target occupancy versus plasma BIIB113 concentrations at the time of the PET scan. The best-fit parameters were used to determine the plasma BIIB113 concentrations that result in 50 % and 90 % of the maximum target occupancy (half-maximal effective concentration [EC_50_] and 90 % effective concentration [EC_90_], respectively).

#### 2.5.3 Exploratory endpoints

Analysis of the concentration-QTc relationship was conducted with BIIB113 plasma concentrations and change from baseline in QTc using Fridericia’s heart rate correction formula (∆QTcF). Concentration-QTc modeling of the relationship between the BIIB113 plasma concentrations, QTc, and other ECG parameters was performed. The change from baseline in cholesterol and 4β-hydroxycholesterol over the study period was assessed using descriptive statistics to assess the effect of BIIB113 on CYP3A activity in the Part B MAD cohorts.

## 3 Results

### 3.1 Participant disposition

In the Part A SAD substudy, 35 participants were screened and enrolled from February 2022 through July 2023 (Fig. 1**A**). A total of 23 participants were randomized to receive BIIB113, and 12 participants received placebo. All 35 participants completed Part A. In the Part B MAD substudy, 27 participants were screened and enrolled from October 2022 through July 2023 (Fig. 1**B**). Eighteen participants were randomized to receive BIIB113, and 9 participants received placebo. A total of 26 participants (96.3 %) completed Part B. One participant in the pooled placebo group discontinued study treatment and was subsequently withdrawn from the study due to an AE of tonsillitis.

In the Part C OGA-PET target occupancy substudy for SAD cohorts (Fig. 1**C**), 7 participants were screened and enrolled from September 2022 through July 2023. All 7 participants were assigned to receive BIIB113, and all participants completed Part C. In the Part C OGA-PET MAD cohort, 3 participants were screened and enrolled from May through July 2023. All 3 participants were assigned to receive BIIB113, and all participants completed Part C.

### 3.2 Baseline demographics

Baseline characteristics of participants from each study arm are summarized in Table 1. In Part A, there were 4 cohorts (randomly assigned to receive either BIIB113 or placebo in a 6:3 ratio). A total of 23 participants were dosed in Cohorts 1 (0.5 mg; *n* = 6), 2 (3 mg; *n* = 6), 3 (15 mg, fasted/fed; *n* = 6), and 4 (50 mg; *n* = 5), with a total of 12 participants in the pooled placebo group. The mean age ± SD was 46.9 ± 13.54 years for the pooled placebo and 46.4 ± 13.10 years for the active BIIB113 dose groups. Of the 23 participants who received BIIB113, 2 (8.7 %) were male, and 21 (91.3 %) were female. Of the 12 participants who received placebo, 2 (16.7 %) were male, and 10 (83.3 %) were female. In Part B, 18 participants were dosed in Cohorts 6 (15 mg; *n* = 6), 7 (50 mg; *n* = 6), and 9 (15 mg [elderly cohort, 65 to 75 years of age]; *n* = 6), with a total of 9 participants in the pooled placebo group. Cohort 8, the highest planned dose cohort (100 mg) in Part B, was not initiated as the dose was refined based on the target occupancy readout from Part C (OGA-PET SAD substudy).

**Table 1:** Characteristics of participants at baseline for the SAD, MAD, and target occupancy studies.

Baseline characteristics	Part A: SAD		Part B: MAD		Part C: target occupancy	
	Pooled placebo (*n* = 12)	Total active (*n* = 23)	Pooled placebo (*n* = 9)	Total active (*n* = 18)	Active only, SAD (*n* = 7)	Active only, MAD (*n* = 3)
**Dose, mg**	–	0.5, 3, 15, 50	–	15, 50	0.5, 3	0.5
**Age in years, mean ± SD**	46.9 ± 13.54	46.4 ± 13.10	46.1 ± 21.00	50.4 ± 16.79	34.7 ± 11.35	32.0 ± 3.61
**Female, n ( %)**	10 (83.3)	21 (91.3)	9 (100.0)	12 (66.7)	7 (100.0)	2 (66.7)
**Weight in kg, mean ± SD**	65.7 ± 10.00	67.5 ± 9.32	70.0 ± 7.53	72.7 ± 11.12	70.0 ± 8.83	64.3 ± 13.60
**BMI in kg/m^2^, mean ± SD**	24.4 ± 2.96	24.7 ± 2.42	26.3 ± 2.56	25.8 ± 2.37	25.5 ± 3.19	23.1 ± 3.55
**Race, n**						
**African American**	–	1	–	1	–	–
**Asian**	1	2	–	–	–	–
**White**	9	16	9	17	7	2
**Not reported**	2	4	–	–	–	1
**Ethnicity, n**						
**Hispanic or Latino**	1	1	–	–	–	–
**Non-Hispanic or Latino**	11	22	9	18	4	3
**Not reported**	–	–	–	–	3	–

BMI, body mass index; MAD, multiple-ascending dose; SAD, single-ascending dose; SD, standard deviation.

The mean age ± SD was 46.1 ± 21.00 years for the pooled placebo and 50.4 ± 16.79 years for the active BIIB113 dose groups. To evaluate the pharmacokinetics, safety, and tolerability of BIIB113 in an older population, Cohort 9, which included participants aged 65 to 75 years, was included and had a median age of 68.5 years (range, 65 to 73 years). Of the 18 participants who received BIIB113, 6 (33.3 %) were male, and 12 (66.7 %) were female. All 9 participants who received placebo were female. Of note, fertile males were excluded from the study as O-GlcNAcylation plays a significant role in sperm motility [23,24]. Toxicology studies revealed comparable effects on spermatogenesis in both rats and dogs in the presence of BIIB113, with full recovery when treatment was stopped (data not published).

In the Part C OGA-PET SAD substudy, there were 2 cohorts: Cohorts 1 (3 mg; *n* = 3) and 2 (0.5 mg; *n* = 4). The mean age ± SD was 34.7 ± 11.35 years for the active BIIB113 dose groups. All 7 participants were female and White. In the Part C OGA-PET MAD substudy, Cohort 5 included 3 participants who received BIIB113 0.5 mg. The mean age ± SD was 32.0 ± 3.61 years for the active BIIB113 dose groups. One participant (33.3 %) was male, and 2 participants (66.7 %) were female. Two participants (66.7 %) were White, and 1 participant (33.3 %) was other (race was not reported).

Overall, the study population included predominantly female, White, and non-Hispanic participants. Demographics and baseline characteristics such as body mass index and weight were similar between the total active dose and the pooled placebo groups (Table 1).

### 3.3 Safety

Table 2 details the safety data from each study arm. Across all study arms, there were no deaths, severe AEs, or SAEs observed. Overall, across study arms, the observed AEs were mild or moderate in intensity. In Part A, AEs were experienced by 11 participants (47.8 %) who received BIIB113 and 5 participants (41.7 %) who received placebo. The most frequently reported (>2 participants in either treatment group) AE was headache (BIIB113: 5 participants [21.7 %]; placebo: 2 participants [16.7 %]). Two participants (8.7 %) who received BIIB113 experienced related AEs (as assessed by the investigator), which included AEs of feces soft, frequent bowel movements, fatigue, and headache; 1 placebo-treated participant (8.3 %) had a related AE (as assessed by the investigator) of headache. No participants discontinued study treatment due to AEs in Part A.

**Table 2:** Summary of safety data.

	Part A: SAD		Part B: MAD		Part C: target occupancy	
	Pooled placebo (*n* = 12)	Total active (*n* = 23)	Pooled placebo (*n* = 9)	Total active (*n* = 18)	Active only SAD (*n* = 7)	Active only MAD (*n* = 3)
**Dose, mg**	–	0.5, 3, 15, 50	–	15, 50	0.5, 3	0.5
**All TEAEs, n ( %)**	5 (41.7)	11 (47.8)	5 (55.6)	13 (72.2)	7 (100.0)	3 (100.0)
**Mild TEAE**	2 (16.7)	8 (34.8)	2 (22.2)	6 (33.3)	6 (85.7)	3 (100.0)
**Moderate TEAE**	3 (25.0)	3 (13.0)	3 (33.3)	7 (38.9)	1 (14.3)	0
**Severe TEAE**	0	0	0	0	0	0
**Related TEAE**	1 (8.3)	2 (8.7)	1 (11.1)	2 (11.1)	0	1 (33.3)
**SAE, n ( %)**	0	0	0	0	0	0
**Deaths, n ( %)**	0	0	0	0	0	0
**Treatment discontinuation due to AE, n ( %)**	0	0	1 (11.1)	1 (5.6)	0	0
**Study withdrawal due to AE, n ( %)**	0	0	1 (11.1)	0	0	0

AE, adverse event; MAD, multiple-ascending dose; SAD, single-ascending dose; SAE, severe adverse event; TEAE, treatment-emergent adverse event.

In Part B, AEs were experienced by 13 participants (72.2 %) who received BIIB113 and 5 participants (55.6 %) who received placebo. The most frequently reported AEs included headache (BIIB113: 6 participants [33.3 %]; placebo: 1 participant [11.1 %]), dizziness (BIIB113: 3 participants [16.7 %]; placebo: 1 participant [11.1 %]), and tremor (BIIB113: 3 participants [16.7 %]; placebo: 0 participants). Two participants (11.1 %) who received BIIB113 experienced related AEs (as assessed by the investigator), which included AEs of tremor and nightmare; 1 placebo-treated participant (11.1 %) experienced a related AE of dizziness. Two participants experienced AEs that led to discontinuation of study treatment: 1 in the MAD Cohort 7 (BIIB113 50-mg) group with tremor (assessed as related to study treatment by the investigator) and 1 in the placebo group with tonsillitis (assessed as unrelated to study treatment by the investigator). The event of tonsillitis also led to the placebo-treated participant withdrawing from the study.

In the Part C OGA-PET SAD/MAD substudy, all participants received BIIB113. In the Part C OGA-PET SAD, 7 participants (100 %) experienced AEs. The most frequently reported AEs included catheter site pain, contusion, and postprocedure contusion (2 participants each [28.6 %]). No AEs in Part C OGA-PET SAD were considered related to study treatment. In the Part C OGA-PET MAD substudy, 3 participants (100 %) experienced AEs. The most frequently reported AE was medical device site eczema (3 participants [100 %]). One participant (33.3 %) reported an AE (headache) that was assessed as related to study treatment by the investigator.

Compared with baseline, there were no clinically meaningful changes in vital signs, laboratory results, or C-SSRS scores. Treatment with BIIB113 was not associated with clinically significant ECG changes over time, and no clinically meaningful trends were observed in any treatment groups. None of the participants had a maximal QTcF interval of >500 milliseconds or an increase in QTcF interval of >60 milliseconds from baseline. Overall, single and multiple doses of BIIB113 were well tolerated up to 50 mg in healthy volunteers.

### 3.4 Pharmacokinetic properties of BIIB113 in the SAD and MAD substudies

**Fig. 2:**
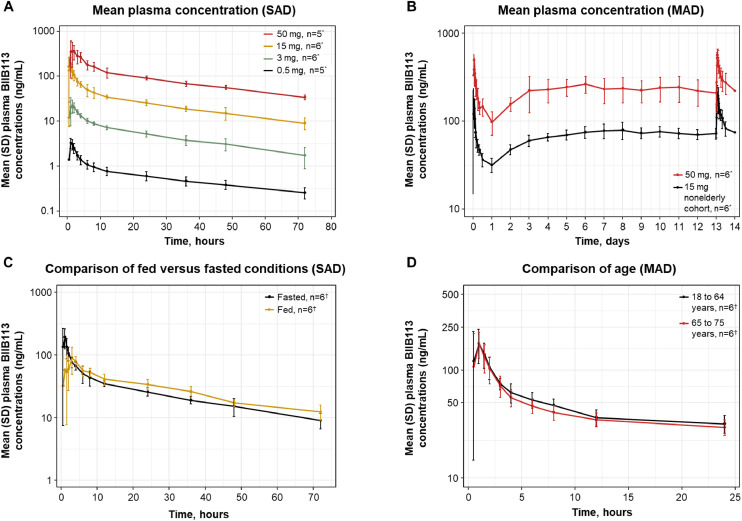
Mean concentration-time profiles from the SAD and MAD cohorts.

The mean BIIB113 concentration-time profiles from the SAD and MAD cohorts are shown in Fig. 2**A** and **B** The pharmacokinetics of BIIB113 demonstrated dose proportionality and steady-state accumulation. The C_max_ and AUC_last_ increased approximately dose proportionally in the SAD and MAD substudies, and the geometric mean half-life (t_1/2_) was approximately 30 h in most cohorts. Additional plasma pharmacokinetic parameters for the SAD and MAD substudies are shown in **Supplemental Tables 1** and **2**.

* Indicates the number of participants with complete plasma BIIB113 concentration-time profiles available. ^†^ Indicates the number of participants with evaluable plasma BIIB113 concentration-time profiles per study arm. MAD, multiple-ascending dose; SAD, single-ascending dose; SD, standard deviation.

Steady state was reached at Day 7, with minimal (approximately 2-fold) accumulation in C_max_ and AUC from 0 to 24 h (AUC_0–24_). Renal clearance was a minor route of elimination for BIIB113 (<5 % of the dose) in the SAD and MAD substudies.

### 3.5 Effect of fasted and fed conditions and age stratification on the pharmacokinetics of BIIB113

The plasma BIIB113 pharmacokinetic results after administration of a single 15-mg oral dose in the fed and fasted states are presented in Fig. 2**C** and **Supplemental Table 3**. Administration of BIIB113 with a high-fat meal had no significant clinical effect on AUC_last_ or AUC_inf_ relative to administration in the fasted state. Following a single oral dose in the fasted state, BIIB113 was rapidly absorbed, with the median time to maximum observed concentration (T_max_) ranging between 1.0 and 1.5 h postdose over the dose range of 0.5 to 50 mg. The geometric mean t_1/2_ of BIIB113 was approximately 30 h in the 1-, 3-, 15-, and 50-mg dose cohorts. Under fasted conditions, BIIB113 systemic exposure increased in an approximately dose-proportional fashion over the dose range of 0.5 to 50 mg. Renal excretion was a minor route of elimination for BIIB113. The administration of BIIB113 with food did not affect the extent of absorption as evidenced by the lack of a relevant change in AUC after administration with or without food; however, the rate of absorption was reduced (50 % decrease in C_max_ and a 2-h increase in T_max_).

The plasma BIIB113 pharmacokinetic profiles in elderly (65 to 75 years of age) and nonelderly (18 to 64 years of age) participants are shown in Fig. 2**D**. Plasma BIIB113 C_max_ and T_max_ values were similar after a single 15-mg dose of BIIB113 administered in a fasted state in the SAD substudy and after the first dose in both of the MAD cohorts (Cohort 9/elderly participants and Cohort 6). The geometric mean plasma BIIB113 pharmacokinetic parameters were generally similar in the MAD cohorts; the geometric mean accumulation ratio on Day 14 for the AUC_tau_ was slightly higher in the elderly (2.61) compared with the nonelderly (1.99) cohort but was not clinically relevant. These results suggest that there are no clinically meaningful differences in the pharmacokinetics of BIIB113 between elderly (65 to 75 years of age) and nonelderly (18 to 64 years of age) participants after single and multiple doses of BIIB113 QD for 14 days.

### 3.6 OGA-PET target occupancy of BIIB113

**Fig. 3:**
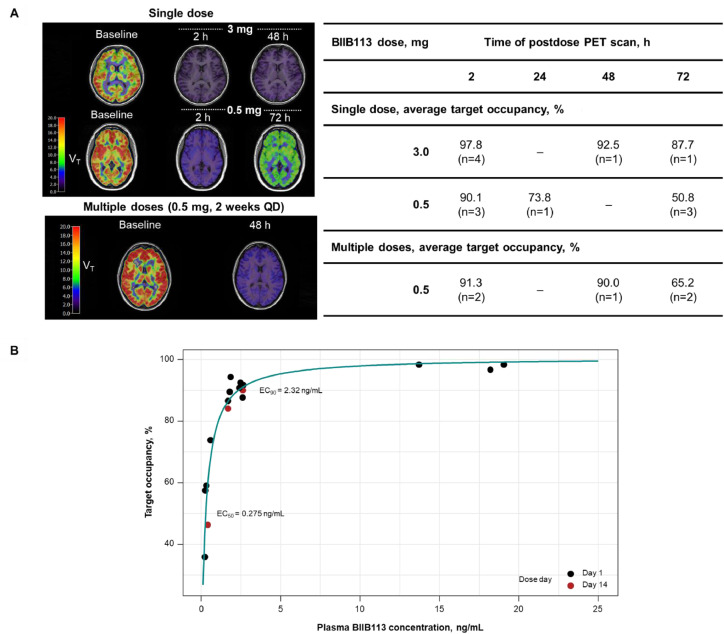
OGA-PET target occupancy and plasma concentration of BIIB113.

The target occupancy of OGA by BIIB113 is presented in Fig. 3**.** Ten participants were dosed with BIIB113 and imaged at various time points after administration (**Supplemental Table 4**). A single dose of BIIB113 3 mg maintained >90 % target occupancy up to 48 h after administration. A single 0.5-mg dose was associated with 90.1 % target occupancy at 2 h after administration. The binding was reversed to approximately 50 % at the 72-h postdose assessment.

The line (panel B) is the fit of an E_max_ model to the observed data. EC_50_, half-maximal effective concentration; EC_90_, 90 % effective concentration; E_max_, maximum effect; OGA, O-GlcNAcase; PET, positron emission tomography; QD, once daily.

Target occupancy was also assessed in a multiple-dose setting in which participants were treated QD for 14 days. Multiple doses of BIIB113 0.5 mg maintained ≥90 % target occupancy for up to 48 h after administration (Fig. 3**A**) and maintained a target occupancy of 65.2 % for up to 72 h after administration. There was no shift in target occupancy following multiple doses of BIIB113.

A simple E_max_ model describes the relationship between plasma BIIB113 concentrations and target occupancy observed on Days 1 and 14 (Fig. 3**B**). There was no apparent delay between the presence of BIIB113 in plasma and target occupancy. The EC_50_ was approximately 0.17 nM (0.0658 ng/mL), and the EC_90_ was 2.32 ng/mL. There was no difference in the relationship on Day 1 and Day 14 QD administration. Thus, BIIB113 0.5 mg QD can achieve a mean target occupancy of ≥90 % over the entire dosing interval.

### 3.7 Cardiodynamic exploratory objectives

BIIB113 had no clinically relevant effect on heart rate or cardiac conduction, defined as the PR or QRS intervals, i.e. the time from the beginning of the P wave (atrial depolarization) to the beginning of the QRS complex (ventricular depolarization). Based on the concentration-QTc analysis, an effect on the placebo-corrected change from baseline in QTcF exceeding 10 ms can be excluded within the full observed range of BIIB113 plasma concentrations up to approximately 900 ng/mL. The information obtained from characterisation using the various tools above, led to the confirmation of the proposed structures for CuAL in Figure 1 and CuGe in Figure 2, respectively.

### 3.8 Assessment of CYP3A activity in the MAD substudy

The absolute median percent changes from baseline on Days 7 and 14 of BIIB113 administration (15 and 50 mg QD) were <20 % for cholesterol and 4β-hydroxycholesterol, consistent with no meaningful induction of CYP3A by BIIB113 at doses up to 50 mg QD. Change from baseline and percent change from baseline in plasma cholesterol and 4β-hydroxycholesterol levels are presented in **Supplemental Tables 5** and **6**.

## 4 Discussion

BIIB113 is an oral small-molecule inhibitor of OGA that had an acceptable safety profile and was well tolerated after single and multiple oral doses up to 50 mg in healthy adult participants in this Phase 1 study. Across all study arms, AEs were mild or moderate in intensity with no deaths, severe AEs, or SAEs observed. The pharmacokinetics of BIIB113 were linear over the dose range studied, with a t_1/2_ of approximately 30 h, demonstrating that QD dosing is appropriate for BIIB113. Renal clearance was a minor route of elimination for BIIB113. The administration of BIIB113 with food did not affect the extent of absorption but decreased the rate of absorption. Furthermore, there were no clinically meaningful differences in the pharmacokinetics of BIIB113 between elderly and nonelderly participants after single and multiple oral doses of BIIB113 15 mg. The elderly population included in this study reflects the age range of the target population in AD. In the OGA-PET substudy, multiple oral QD doses as low as BIIB113 0.5 mg maintained a target occupancy of ≥90 % up to 48 h after last dose administration. BIIB113 had no clinically relevant effect on cardiovascular function. There were no significant increases in levels of 4β-hydroxycholesterol observed over the dose range of 15 to 50 mg QD, which suggests that there was no induction of CYP3A by BIIB113.

Results emanating from the use of the OGA-PET tracer in the present study suggest its importance as a clinical development tool for dose selection in future studies. However, it has yet to be determined whether OGA inhibition is beneficial for the treatment of AD and other tauopathies.

OGA is a highly ubiquitous enzyme [25], and long-term exposure to OGA inhibitors, traditionally 18 months in clinical trials of patients with AD, are needed to understand its safety profile and efficacy. Ceperognastat (LY3372689) was the first small-molecule OGA inhibitor to advance to a Phase 2 clinical trial [26]. Ceperognastat failed to demonstrate benefit in clinical endpoints despite high levels of target engagement in patients with AD who met the inclusion criteria of low-medium and high baseline tau PET burden. Of note, in the low-medium tau burden group, the high-dose group performed worse on the primary endpoint, whereas the low-dose group showed a trend towards improvement compared with the placebo group. On the other hand, volumetric magnetic resonance imaging changes indicated less brain volume loss in the treatment arms, and tau PET and fluid biomarker changes suggested a treatment effect on tau pathology and neuroinflammation, in alignment with findings in model systems of preclinical disease. The lack of clinical benefit despite evidence of biomarker changes may indicate that the biomarker changes were not robust enough to impact the progression of the disease in the patient population studied. Furthermore, many proteins are altered by O-GlcNAcylation; thus, OGA inhibition may have elicited pathway responses that negatively impacted clinical outcome measures. For example, a recent study described synaptic toxicity associated with OGA inhibition [27]. The pleiotropic nature of O-GlcNAc biology poses a potential challenge to develop OGA inhibitors for the treatment of chronic neurodegenerative diseases. Current studies of OGA inhibition offer the opportunity to learn more about the effect of OGA inhibition on measures of clinical efficacy, biomarkers, and safety.

### 4.1 Study limitations

This first-in-human study of BIIB113 has a relatively small sample size, as is typical of Phase 1 studies, and it was conducted in healthy volunteers for a short duration (14 days) in the MAD substudy. In this study, BIIB113 0.5 mg QD achieved ≥90 % target occupancy of OGA over the dosing interval studies. Whether this high occupancy of the OGA enzyme by BIIB113 could have effects on tau measured by tau PET or other tau biomarkers will require evaluation in a larger clinical study in patients with AD. Additionally, the exclusion of fertile male participants may have influenced the female:male ratio in the study cohorts as a higher number of female participants were enrolled in all cohorts.

## 5 Conclusions

In this Phase 1 trial, BIIB113 had an acceptable safety profile and was well tolerated by healthy adult participants after single and multiple oral doses up to 50 mg, and there were no clinically meaningful differences in the pharmacokinetics of BIIB113 between elderly and nonelderly participants. Multiple 0.5-mg doses of BIIB113 maintained OGA target occupancy of ≥90 % up to 48 h after the last dose administration.

## Declaration of generative AI and AI-assisted technologies in the writing process

The authors confirm that they have not used AI or AI-assisted technologies in the writing process or in the preparation of this manuscript.

## Funding

This study was funded by Biogen, Inc. Biogen authors were involved in the design and conduct of the study as well as the collection, analysis, and interpretation of data. Biogen authors prepared, reviewed, and approved the manuscript.

## CRediT authorship contribution statement

**Flavia C. Nery:** Writing – review & editing, Writing – original draft, Visualization, Validation, Supervision, Methodology, Investigation, Funding acquisition, Data curation, Conceptualization. **Maciej Kaliszczak:** Writing – review & editing, Writing – original draft, Visualization, Validation, Supervision, Software, Resources, Project administration, Methodology, Investigation, Funding acquisition, Formal analysis, Data curation, Conceptualization. **Ben Suttle:** Writing – review & editing, Writing – original draft, Validation, Methodology, Investigation, Data curation, Conceptualization. **Lori Jones:** Writing – review & editing. **Shuang Wu:** Writing – review & editing, Supervision, Methodology. **Jing Xie:** Writing – review & editing, Formal analysis, Data curation. **Gioacchino Curiale:** Writing – review & editing. **Esin Yesilalan:** Writing – review & editing. **Beth Hirschhorn:** Writing – review & editing. **Denisa Wilkes:** Writing – review & editing, Investigation. **Dave Singh:** Writing – review & editing, Supervision, Investigation. **Martin Bolin:** Writing – review & editing, Methodology, Investigation, Data curation. **Sangram Nag:** Writing – review & editing, Validation, Methodology. **Andrea Varrone:** Writing – review & editing. **Per Stenkrona:** Supervision, Project administration, Investigation, Conceptualization. **Anton Forsberg Morén:** Writing – review & editing, Validation, Supervision, Project administration, Methodology, Investigation, Data curation, Conceptualization. **Christer Halldin:** Writing – review & editing, Supervision, Resources, Project administration, Methodology, Funding acquisition, Data curation, Conceptualization. **Jeffrey Yachnin:** Investigation. **H. Moore Arnold:** Writing – review & editing, Supervision, Resources, Project administration. **Szofia Bullain:** Writing – review & editing, Supervision, Resources. **Jaren Landen:** Writing – review & editing, Writing – original draft, Supervision, Investigation, Formal analysis, Conceptualization. **Diana Gallagher:** Writing – review & editing, Supervision, Resources, Funding acquisition. **Heike Hering:** Writing – review & editing, Writing – original draft, Conceptualization.

## Declaration of competing interest

The authors declare the following financial interests/personal relationships that may be considered as potential competing interests: Flavia C. Nery reports a relationship with Biogen, Inc that includes employment and equity or stocks. Shuang Wu reports a relationship with Biogen, Inc that includes employment and equity or stocks. Jing Xie reports a relationship with Biogen, Inc that includes employment and equity or stocks. Gioacchino G. Curiale reports a relationship with Biogen, Inc that includes employment and equity or stocks. Esin Yesilalan reports a relationship with Biogen, Inc that includes employment and equity or stocks. Beth Hirschhorn reports a relationship with Biogen, Inc that includes employment and equity or stocks. H. Moore Arnold reports a relationship with Biogen, Inc that includes employment and equity or stocks. Szofia Bullain reports a relationship with Biogen, Inc that includes employment and equity or stocks. Diana Gallagher reports a relationship with Biogen Inc that includes employment and equity or stocks. Heike Hering reports a relationship with Biogen, Inc that includes employment and equity or stocks. Maciej Kaliszczak reports a relationship with Biogen, Inc that includes former employment and equity or stocks. Jaren Landen reports a relationship with Biogen, Inc that includes former employment and equity or stocks. Ben Suttle reports a relationship with Biogen, Inc that includes consulting or an advisory role. Dave Singh reports a relationship with Aerogen that includes consulting or an advisory role. Dave Singh reports a relationship with AstraZeneca that includes: consulting or advisory. Dave Singh reports a relationship with Boehringer Ingelheim that includes: consulting or advisory. Dave Singh reports a relationship with Chiesi Pharmaceuticals Inc that includes: consulting or advisory. Dave Singh reports a relationship with Cipla that includes: consulting or advisory. Dave Singh reports a relationship with CSL Behring LLC that includes: consulting or advisory. Dave Singh reports a relationship with EpiEndo that includes: consulting or advisory. Dave Singh reports a relationship with Genen tech that includes: consulting or advisory. Dave Singh reports a relationship with GSK that includes: consulting or advisory. Dave Singh reports a relationship with Glenmark Pharmaceuticals Limited that includes: consulting or advisory. Dave Singh reports a relationship with Gossamer Bio Inc that includes: consulting or advisory. Dave Singh reports a relationship with Kinaset Therapeutics that includes: consulting or advisory Dave Singh reports a relationship with Menarini that includes: consulting or advisory. Dave Singh reports a relationship with Novartis Pharmaceuticals Corporation that includes: consulting or advisory. Dave Singh reports a relationship with Orion Corporation that includes: consulting or advisory. Dave Singh reports a relationship with PULMATRiX Inc that includes: consulting or advisory. Dave Singh reports a relationship with Sanofi that includes: consulting or advisory. Dave Singh reports a relationship with Teva Pharmaceuticals Industries Inc that includes: consulting or advisory. Dave Singh reports a relationship with Theravance Biopharma Inc that includes: consulting or advisory. Dave Singh reports a relationship with Verona Pharma that includes: consulting or advisory. All other authors declare that they have no known competing financial interests or personal relationships that could have appeared to influence the work reported in this paper.

## Appendix Supplementary materials

Image, application 1

## References

[1] DeTure M.A., Dickson D.W. (2019). The neuropathological diagnosis of Alzheimer's disease. Mol Neurodegener.

[2] Long J.M., Holtzman D.M. (2019). Alzheimer disease: an update on pathobiology and treatment strategies. Cell.

[3] Braak H., Braak E. (1991). Neuropathological stageing of Alzheimer-related changes. Acta Neuropathol.

[4] Nelson P.T., Alafuzoff I., Bigio E.H. (2012). Correlation of Alzheimer disease neuropathologic changes with cognitive status: a review of the literature. J Neuropathol Exp Neurol.

[5] Zhu Y., Shan X., Yuzwa S.A., Vocadlo D.J. (2014). The emerging link between O-GlcNAc and Alzheimer disease. J Biol Chem.

[6] Hammes J., Bischor G.N., Drzezga A. (2017). Molecular imaging in early diagnosis, differential diagnosis and follow-up of patients with neurodegenerative diseases. Clin Transl Imaging.

[7] Bischof G.N., Jessen F., Fliessbach K. (2016). Impact of tau and amyloid burden on glucose metabolism in Alzheimer's disease. Ann Clin Transl Neurol.

[8] Bond M.R., Hanover J.A. (2015). A little sugar goes a long way: the cell biology of O-GlcNAc. J Cell Biol.

[9] Paneque A., Fortus H., Zheng J., Werlen G., Jacinto E. (2023). The hexosamine biosynthesis pathway: regulation and function. Genes (Basel).

[10] Cook B.E., Nag S., Arakawa R. (2023). Development of a PET tracer for OGA with improved kinetics in the living brain. J Nucl Med.

[11] Yuzwa S.A., Shan X., Macauley M.S. (2012). Increasing O-GlcNAc slows neurodegeneration and stabilizes tau against aggregation. Nat Chem Biol.

[12] Graham D.L., Gray A.J., Joyce J.A. (2014). Increased O-GlcNAcylation reduces pathological tau without affecting its normal phosphorylation in a mouse model of tauopathy. Neuropharmacology.

[13] Borghgraef P., Menuet C., Theunis C. (2013). Increasing brain protein O-GlcNAc-ylation mitigates breathing defects and mortality of Tau.P301L mice. PLoS One.

[14] Permanne B., Sand A., Ousson S. (2022). O-GlcNAcase inhibitor ASN90 is a multimodal drug candidate for tau and α-synuclein proteinopathies. ACS Chem Neurosci.

[15] Wang X., Smith K., Pearson M. (2018). Early intervention of tau pathology prevents behavioral changes in the rTg4510 mouse model of tauopathy. PLoS One.

[16] Hastings N.B., Wang X., Song L. (2017). Inhibition of O-GlcNAcase leads to elevation of O-GlcNAc tau and reduction of tauopathy and cerebrospinal fluid tau in rTg4510 mice. Mol Neurodegener.

[17] Yuzwa S.A., Shan X., Jones B.A. (2014). Pharmacological inhibition of O-GlcNAcase (OGA) prevents cognitive decline and amyloid plaque formation in bigenic tau/APP mutant mice. Mol Neurodegener.

[18] Nery F.C., Hering H., Jung Y. (2024). Results of the first-in-human, randomized, blinded, placebo-controlled, single- and multiple- ascending dose study of BIIB113 in healthy volunteers. AD/PD.

[19] Nag S., Bolin M., Datta P. (2023). Development of a novel [^11^C]CO-labeled positron emission tomography radioligand [^11^C]BIO-1819578 for the detection of O-GlcNAcase enzyme activity. ACS Chem Neurosci.

[20] ClinicalTrials.gov. A study to evaluate the safety, tolerability, and pharmacokinetics, with target occupancy study of BIIB113 in healthy participants. https://clinicaltrials.gov/study/NCT05195008. Accessed June 10, 2024.

[21] Moein M.M., Nakao R., Amini N. (2019). Sample preparation techniques for radiometabolite analysis of positron emission tomography radioligands; trends, progress, limitations and future prospects. TrAC Trends Anal Chem.

[22] Ichise M., Toyama H., Innis R.B., Carson R.E. (2002). Strategies to improve neuroreceptor parameter estimation by linear regression analysis. J Cereb Blood Flow Metab.

[23] Konzman D., Fukushige T., Dagnachew M., Krause M., Hanover J.A. (2022). O-GlcNAc transferase plays a non-catalytic role in C. elegans male fertility. PLoS Genet.

[24] Qian Z., Li C., Zhao S. (2023). Age-related elevation of O-GlcNAc causes meiotic arrest in male mice. Cell Death Discov.

[25] Bartolomé-Nebreda J.M., Trabanco A.A., Velter A.I., Buijnsters P. (2021). O-GlcNAcase inhibitors as potential therapeutics for the treatment of Alzheimer's disease and related tauopathies: analysis of the patent literature. Expert Opin Ther Pat.

[26] Kielbasa W., Goldsmith P., Donnelly K.B. (2024). Discovery and clinical translation of ceperognastat, an O-GlcNAcase (OGA) inhibitor, for the treatment of Alzheimer’s disease. Alzheimers Dement (N Y).

[27] Meade J., Mesa H., Liu L., Zhang Q. (2025). Synaptic toxicity of OGA inhibitors and the failure of ceperognastat. bioRxiv.

