# Erratum to “Results of the first-in-human, randomized, double-blind, placebo-controlled, single- and multiple-ascending dose study of BIIB113 in healthy volunteers” [The Journal of Prevention of Alzheimer's Disease volume 12 (2025) 100302]

**DOI:** 10.1016/j.tjpad.2025.100425

**Published:** 2025-11-17

**Authors:** Flavia C. Nery, Maciej Kaliszczak, Ben Suttle, Lori Jones, Shuang Wu, Jing Xie, Gioacchino Curiale, Esin Yesilalan, Beth Hirschhorn, Denisa Wilkes, Dave Singh, Martin Bolin, Sangram Nag, Andrea Varrone, Per Stenkrona, Anton Forsberg Morén, Christer Halldin, Jeffrey Yachnin, H. Moore Arnold, Szofia Bullain, Jaren Landen, Diana Gallagher, Heike Hering

**Affiliations:** a Biogen, Cambridge, MA, USA; b Biogen, Durham, NC, USA; c qPharmetra, Raleigh, NC, USA; d Hammersmith Medicines Research Limited, London, UK; e Medicines Evaluation Unit, The Langley Building, Manchester University NHS Foundation Trust, University of Manchester, Manchester, UK; f Department of Clinical Neuroscience, Center for Psychiatry Research, Karolinska Institutet, Stockholm, Sweden; g Karolinska Comprehensive Cancer Center, Huddinge, Sweden

The publisher regret the insertion by error of the following sentence page 7 at the end of part 3.7: “The information obtained from characterisation using the various tools above, led to the confirmation of the proposed structures for CuAL in Figure 1 and CuGe in Figure 2, respectively.”

The publisher would like to apologise for any inconvenience caused.

