# Supplementary material for: Results of the first-in-human, randomized, double-blind, placebo-controlled, single- and multiple-ascending dose study of BIIB113 in healthy volunteers
Source: J Prev Alzheimers Dis. 2025 Jul 21;12(8):100302. doi: 10.1016/j.tjpad.2025.100302 (PMC12413702; doi:10.1016/j.tjpad.2025.100302)
Supplement: Supplementary file 1 [file mmc1.docx]

**Supplement**

**Supplemental Figure 1. O-GlcNAcylation links glucose hypometabolism with NFT formation**

**
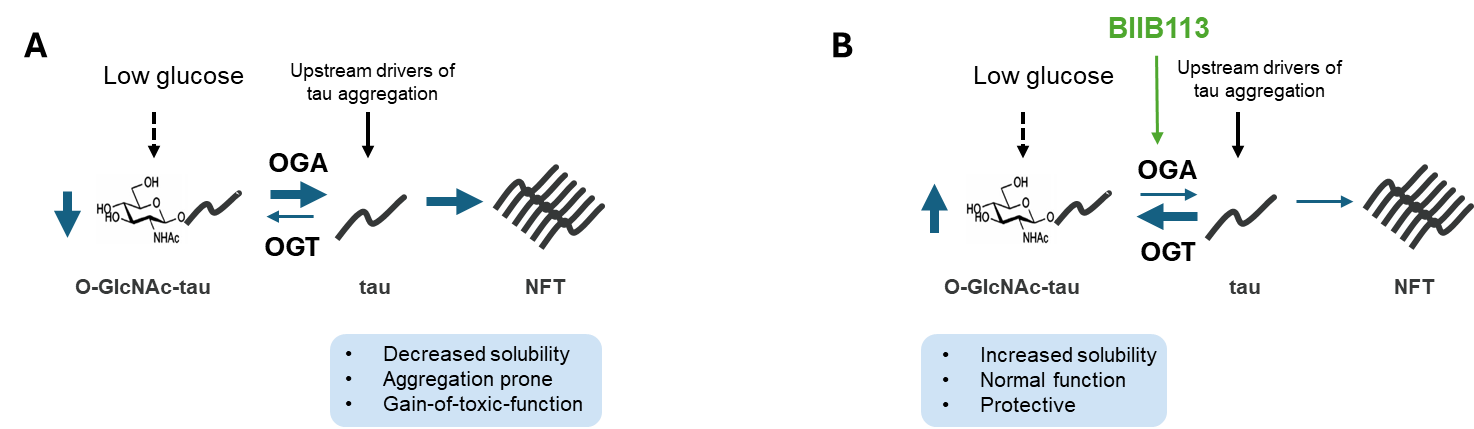
**

(A) Low glucose availability results in low levels of O-GlcNAcylated tau in neurons, making tau more susceptible to NFT formation by upstream drivers of tau aggregation. (B) Inhibition of OGA by BIIB113 increases levels of O-GlcNAcylated tau, protecting it from aggregation. NFT, neurofibrillary tangle; OGA, O-GlcNAcase; O-GlcNAc, O-linked N-acetylglucosamine; OGT, O-GlcNAc transferase.

**Supplemental Table 1. Plasma pharmacokinetic parameters of BIIB113 in the SAD substudy**

|  | **Cohort 1**  **0.5 mg**  **(n=6)** | **Cohort 2**  **3 mg**  **(n=6)** | **Cohort 3**  **15 mg**  **Period 1**  **(n=6)** | **Cohort 3**  **15 mg**  **Period 2**  **(n=6)** | **Cohort 4**  **50 mg**  **(n=5)** |
| --- | --- | --- | --- | --- | --- |
| **C_max_ geometric mean, ng/mL** | 3.91 | 25.36 | 203.62 | 102.88 | 503.82 |
| **C_max_, geometric CV, %** | 27.2 | 35.5 | 45.2 | 38.9 | 42.3 |
| **AUC_last_, geometric mean, h*ng/mL** | 42.93 | 360.25 | 1873.76 | 2062.51 | 6464.24 |
| **AUC_last_, geometric CV, %** | 20.2 | 17.4 | 19.4 | 17.3 | 10.5 |
| **AUC_inf_, geometric mean, h*ng/mL** | 38.76 | 394.96 | 2163.38 | 2472.11 | 8064.06 |
| **AUC_inf_, geometric CV, %** | N/A | 17.6 | 23.4 | 17.0 | 5.7 |
| **t_1/2_, geometric mean, hours** | 39.76 | 29.11 | 31.31 | 31.51 | 31.74 |
| **t_1/2_, geometric CV, %** | 33.2 | 41.2 | 21.2 | 9.5 | 22.0 |
| **T_max_, median (min, max)** | 1.02 (0.50, 1.53) | 1.39 (0.50, 3.02) | 1.00 (0.55, 1.03) | 3.00 (1.00, 4.00) | 1.50 (0.52, 3.00) |

AUC_inf_, area under the concentration-time curve from time zero extrapolated to infinity; AUC_last_, area under the concentration-time curve from time zero to the time of the last measurable concentration; C_max_, maximum observed concentration; CV, coefficient of variation; N/A, not available; SAD, single-ascending dose; t_1/2_, half-life; T_max_, time to maximum observed concentration.

**Supplemental Table 2. Plasma pharmacokinetic parameters of BIIB113 in the MAD substudy**

|  | **Cohort 6**  **15 mg**  **(n=6)** | **Cohort 7**  **50 mg**  **(n=6)** | **Cohort 9/Elderly**  **15 mg**  **(n=6)** |
| --- | --- | --- | --- |
| **C_max_ geometric mean, ng/mL** |  |  |  |
| **Day 1** | 215.73 | 595.83 | 203.33 |
| **Day 14** | 239.44 | 590.66 | 258.00 |
| **C_max_, geometric CV, %** |  |  |  |
| **Day 1** | 20.4 | 26.8 | 14.3 |
| **Day 14** | 18.8 | 13.2 | 16.5 |
| **AUC_tau_, geometric mean, h*ng/mL** |  |  |  |
| **Day 1** | 1159.99 | 3731.87 | 1031.13 |
| **Day 14** | 2434.71 | 6968.30 | 2688.62 |
| **AUC_tau_, geometric CV, %** |  |  |  |
| **Day 1** | 16.4 | 9.6 | 16.1 |
| **Day 14** | 12.9 | 25.1 | 18.7 |
| **T_max_, median (min, max), hours** |  |  |  |
| **Day 1** | 1.00 (0.50, 1.53) | 1.01 (0.50, 1.50) | 1.00 (0.50, 1.50) |
| **Day 14** | 1.51 (1.00, 3.00) | 1.50 (1.00, 1.50) | 1.00 (0.50, 1.72) |

AUC_tau_, area under the concentration-time curve within a dosing interval; C_max_, maximum observed concentration; CV, coefficient of variation; MAD, multiple-ascending dose, T_max_, time to maximum observed concentration.

**Supplemental Table 3. Effect of BIIB113 in fed and fasted states in the SAD substudy**

| **Cohort 3** | | |
| --- | --- | --- |
|  | **Period 1**  **BIIB113 15 mg, fasted**  **(n=6)** | **Period 2**  **BIIB113 15 mg, fed**  **(n=6)** |
| **C_max_, geometric mean ratio**  **(90% CI)** | - | 0.505 (0.337-0.757) |
| **AUC_inf_, geometric mean ratio**  **(90% CI)** | - | 0.992 (0.987-0.997) |

Geometric mean ratios are the ratio of fed dose/fasted dose. The CI for the difference in the least squares mean estimates of the ln-transformed values (fed/fasted) was obtained from the mixed model. Antilogs of the confidence limits were taken to construct the CI for the geometric mean ratio. AUC_inf_, area under the concentration-time curve from time zero extrapolated to infinity; CI, confidence interval; C_max_, maximum observed concentration; SAD, single-ascending dose.

**Supplemental Table 4. Characteristics of participants and TO from the OGA-PET substudies**

| **Cohort** | **Participant number** | **Sex** | **Age** | **Weight, kg** | **PET visit number** | **TO, %** |
| --- | --- | --- | --- | --- | --- | --- |
| **SAD (0.5 mg/kg)** | 1 | Female | 20 | 59 | 1 | N/A |
|  |  |  |  | 59 | 2 | 98.4 |
|  |  |  |  | 58 | 3 | 92.5 |
|  | 2 | Female | 49 | 80 | 1 | N/A |
|  |  |  |  | 80 | 2 | 96.7 |
|  |  |  |  | – | N/A* | N/A* |
|  | 3 | Female | 30 | 70 | 1 | N/A |
|  |  |  |  | 70 | 2 | 98.4 |
|  |  |  |  | 69 | 3 | 87.7 |
| **SAD (0.5 mg/kg)** | 4 | Female | 39 | 78 | 1 | N/A |
|  |  |  |  | 78 | 2 | 89.5 |
|  |  |  |  | 78 | 3 | 59.1 |
|  | 5 | Female | 25 | 63 | 1 | N/A |
|  |  |  |  | 63 | 2 | 94.3 |
|  |  |  |  | 64 | 3 | 57.5 |
|  | 6 | Female | 31 | 64 | 1 | N/A |
|  |  |  |  | – | N/A* | N/A* |
|  |  |  |  | 64 | 3 | 35.9 |
|  | 7 | Female | 49 | 82 | 1 | N/A |
|  |  |  |  | 82 | 2 | 86.6 |
|  |  |  |  | 81 | 3 | 73.8 |
|  | 8 | Male | 36 | 80 | 1 | N/A |
|  |  |  |  | 80 | 2 | 91.7 |
|  |  |  |  | 80 | 3 | 84.1 |
| **MAD (0.5 mg/kg)** | 9 | Female | 31 | 61 | 1 | N/A |
|  |  |  |  | – | N/A* | N/A* |
|  |  |  |  | 62 | 3 | 90 |
|  | 10 | Female | 29 | 52 | 1 | N/A |
|  |  |  |  | 52 | 2 | 90.8 |
|  |  |  |  | 52 | 3 | 46.3 |

*Not available (N/A) due to radiochemistry technical issues. MAD, multiple-ascending dose; N/A, not available; OGA, O-GlcNAcase; PET, positron emission tomography; SAD, single-ascending dose; TO, target occupancy.

**Supplemental Table 5. Change from baseline in cholesterol plasma levels in the MAD substudy**

|  | **Pooled placebo**  **(n=9)** | **Cohort 6**  **15 mg (n=6)** | **Cohort 7**  **50 mg (n=6)** | **Cohort 9/Elderly**  **15 mg (n=6)** | **Total active**  **(n=18)** |
| --- | --- | --- | --- | --- | --- |
| **Baseline** | | | | | |
| **n** | 9 | 6 | 6 | 6 | 18 |
| **Mean (SD), mg/mL** | 2.117 (0.3956) | 1.952 (0.5732) | 1.975 (0.2702) | 2.210 (0.1604) | 2.046 (0.3743) |
| **Median, mg/mL** | 2.080 | 1.880 | 2.055 | 2.220 | 2.100 |
| **Min, Max, mg/mL** | 1.60, 2.98 | 1.28, 2.92 | 1.45, 2.17 | 1.97, 2.41 | 1.28, 2.92 |
| **CV, %** | 18.7 | 29.4 | 13.7 | 7.3 | 18.3 |
|  | | | | | |
| **Predose day 7** | | | | | |
| **n** | 8 | 6 | 6 | 6 | 18 |
| **Mean (SD), mg/mL** | 2.064 (0.3945) | 2.028 (0.5901) | 1.863 (0.3956) | 2.487 (0.3853) | 2.126 (0.5155) |
| **Median, mg/mL** | 2.170 | 1.865 | 1.955 | 2.480 | 2.110 |
| **Min, Max, mg/mL** | 1.57, 2.57 | 1.47, 3.08 | 1.29, 2.33 | 2.05, 2.95 | 1.29, 3.08 |
| **CV, %** | 19.1 | 29.1 | 21.2 | 15.5 | 24.2 |
|  | | | | | |
| **Change from baseline to predose day 7** | | | | | |
| **n** | 8 | 6 | 6 | 6 | 18 |
| **Mean (SD), mg/mL** | -0.118 (0.5409) | 0.077 (0.3147) | -0.112 (0.2561) | 0.277 (0.3429) | 0.081 (0.3311) |
| **Median, mg/mL** | -0.080 | 0.085 | -0.100 | 0.280 | 0.050 |
| **Min, Max, mg/mL** | -1.26, 0.49 | -0.26, 0.59 | -0.43, 0.23 | -0.14, 0.76 | -0.43, 0.76 |
| **CV, %** | -460.3 | 410.5 | -229.3 | 123.9 | 411.0 |
|  | | | | | |
| **% Change from baseline to predose day 7** | | | | | |
| **n** | 8 | 6 | 6 | 6 | 18 |
| **Mean (SD)** | -3.669 (20.8813) | 5.376 (17.5341) | -5.997 (12.6957) | 12.513 (15.3996) | 3.964 (16.4043) |
| **Median** | -4.546 | 3.056 | -6.443 | 12.591 | 2.555 |
| **Min, Max** | -42.28, 23.56 | -11.98, 34.50 | -21.94, 10.95 | -5.98, 34.70 | -21.94, 34.70 |
| **CV, %** | -569.2 | 326.1 | -211.7 | 123.1 | 413.8 |
|  |  |  |  |  |  |
| **Predose day 14** |  |  |  |  |  |
| **n** | 8 | 6 | 6 | 6 | 18 |
| **Mean (SD), mg/mL** | 1.958 (0.3351) | 2.135 (0.5947) | 1.873 (0.3101) | 2.223 (0.3996) | 2.077 (0.4502) |
| **Median, mg/mL** | 1.990 | 2.185 | 1.870 | 2.180 | 2.075 |
| **Min, Max, mg/mL** | 1.46, 2.36 | 1.42, 3.09 | 1.52, 2.39 | 1.72, 2.73 | 1.42, 3.09 |
| **CV, %** | 17.1 | 27.9 | 16.6 | 18.0 | 21.7 |
|  |  |  |  |  |  |
| **Change from baseline to predose day 14** | | | | | |
| **n** | 8 | 6 | 6 | 6 | 18 |
| **Mean (SD), mg/mL** | -0.224 (0.5108) | 0.183 (0.4594) | -0.102 (0.2398) | 0.013 (0.3916) | 0.032 (0.3723) |
| **Median, mg/mL** | -0.035 | 0.240 | -0.170 | -0.075 | 0.055 |
| **Min, Max, mg/mL** | -1.11, 0.22 | -0.63, 0.63 | -0.36, 0.29 | -0.45, 0.54 | -0.63, 0.63 |
| **CV, %** | -228.3 | 250.6 | -235.9 | 2937.3 | 1175.7 |
|  |  |  |  |  |  |
| **% Change from baseline to predose day 14** | | | | | |
| **n** | 8 | 6 | 6 | 6 | 18 |
| **Mean (SD)** | -8.289 (20.3965) | 12.451 (25.8575) | -4.748 (11.8706) | 0.723 (17.5600) | 2.809 (19.5777) |
| **Median** | -1.285 | 15.020 | -8.128 | -2.867 | 2.826 |
| **Min, Max** | -37.25, 10.28 | -30.73, 36.84 | -18.37, 13.81 | -19.23, 24.66 | -30.73, 36.84 |
| **CV, %** | -246.1 | 207.7 | -250.0 | 2430.1 | 697.1 |

CV, coefficient of variation; MAD, multiple-ascending dose; Max, maximum; Min, minimum; SD, standard deviation. Pooled placebo represents all participants from the MAD substudy who received placebo.

**Supplemental Table 6. Change from baseline in 4β-hydroxycholesterol plasma levels in the MAD substudy**

|  | **Pooled placebo**  **(n=9)** | **Cohort 6**  **15 mg (n=6)** | **Cohort 7**  **50 mg (n=6)** | **Cohort 9/Elderly**  **15 mg (n=6)** | **Total active**  **(n=18)** |
| --- | --- | --- | --- | --- | --- |
| **Baseline** | | | | | |
| **n** | 9 | 6 | 6 | 6 | 18 |
| **Mean (SD), ng/mL** | 32.611 (10.2067) | 28.833 (9.4794) | 39.467 (13.3606) | 29.267 (6.6422) | 32.522 (10.8384) |
| **Median, ng/mL** | 34.300 | 25.050 | 37.350 | 28.500 | 29.450 |
| **Min, Max, ng/mL** | 19.30, 48.50 | 21.60, 46.70 | 24.10, 59.00 | 20.30, 39.90 | 20.30, 59.00 |
| **CV, %** | 31.3 | 32.9 | 33.9 | 22.7 | 33.3 |
|  | | | | | |
| **Predose day 7** | | | | | |
| **n** | 8 | 6 | 6 | 6 | 18 |
| **Mean (SD), ng/mL** | 32.550 (11.7943) | 28.817 (7.3303) | 38.033 (11.7089) | 31.667 (7.6154) | 32.839 (9.4288) |
| **Median, ng/mL** | 32.750 | 27.700 | 34.600 | 31.900 | 30.850 |
| **Min, Max, ng/mL** | 17.60, 52.90 | 20.80, 41.10 | 25.60, 54.20 | 20.70, 41.30 | 20.70, 54.20 |
| **CV, %** | 36.2 | 25.4 | 30.8 | 24.0 | 28.7 |
|  | | | | | |
| **Change from baseline to predose day 7** | | | | | |
| **n** | 8 | 6 | 6 | 6 | 18 |
| **Mean (SD), ng/mL** | 0.238 (2.6827) | -0.017 (3.5414) | -1.433 (3.1741) | 2.400 (5.2790) | 0.317 (4.1833) |
| **Median, ng/mL** | -0.400 | -0.100 | -0.300 | 0.900 | 0.400 |
| **Min, Max, ng/mL** | -3.20, 4.40 | -5.60, 3.90 | -5.90, 1.50 | -2.60, 12.60 | -5.90, 12.60 |
| **CV, %** | 1129.6 | -21248.5 | -221.4 | 220.0 | 1321.1 |
|  | | | | | |
| **% Change from baseline to predose day 7** | | | | | |
| **n** | 8 | 6 | 6 | 6 | 18 |
| **Mean (SD)** | 0.352 (9.0320) | 1.722 (11.6470) | -2.458 (7.2765) | 9.119 (20.4937) | 2.794 (14.2569) |
| **Median** | -0.667 | -0.914 | -0.992 | 2.740 | 1.595 |
| **Min, Max** | -9.22, 15.24 | -11.99, 17.26 | -13.32, 6.22 | -9.09, 49.22 | -13.32, 49.22 |
| **CV, %** | 2564.6 | 676.3 | -296.0 | 224.7 | 510.2 |
|  |  |  |  |  |  |
| **Predose day 14** |  |  |  |  |  |
| **n** | 6 | 6 | 6 | 6 | 18 |
| **Mean (SD), ng/mL** | 29.750 (9.7566) | 27.550 (8.6572) | 39.700 (12.4045) | 28.450 (5.7927) | 31.900 (10.4652) |
| **Median, ng/mL** | 31.150 | 25.100 | 36.750 | 28.550 | 30.050 |
| **Min, Max, ng/mL** | 15.80, 43.60 | 19.80, 40.40 | 26.40, 60.20 | 18.90, 36.40 | 18.90, 60.20 |
| **CV, %** | 32.8 | 31.4 | 31.2 | 20.4 | 32.8 |
|  |  |  |  |  |  |
| **Change from baseline to predose day 14** | | | | | |
| **n** | 6 | 6 | 6 | 6 | 18 |
| **Mean (SD), ng/mL** | -3.717 (3.9656) | -1.283 (3.9311) | 0.233 (4.9690) | -0.817 (2.6026) | -0.622 (3.7717) |
| **Median, ng/mL** | -3.400 | -1.750 | 0.600 | -1.450 | -1.250 |
| **Min, Max, ng/mL** | -10.60, 1.00 | -6.30, 3.90 | -8.20, 7.00 | -3.50, 4.20 | -8.20, 7.00 |
| **CV, %** | -106.7 | -306.3 | 2129.6 | -318.7 | -606.2 |
|  |  |  |  |  |  |
| **% Change from baseline to predose day 14** | | | | | |
| **n** | 6 | 6 | 6 | 6 | 18 |
| **Mean (SD)** | -9.923 (10.0999) | -3.947 (12.9125) | 2.374 (13.6941) | -2.217 (9.2886) | -1.263 (11.7091) |
| **Median** | -9.862 | -7.928 | 1.017 | -5.084 | -4.223 |
| **Min, Max** | -23.35, 4.76 | -17.81, 15.35 | -18.51, 23.03 | -8.77, 16.41 | -18.51, 23.03 |
| **CV, %** | -101.8 | -327.2 | 576.8 | -418.9 | -926.9 |

CV, coefficient of variation; MAD, multiple-ascending dose; Max, maximum; Min, minimum; SD, standard deviation. Pooled placebo represents all participants from the MAD substudy who received placebo.
